# Event-Guided Image Super-Resolution Reconstruction

**DOI:** 10.3390/s23042155

**Published:** 2023-02-14

**Authors:** Guangsha Guo, Yang Feng, Hengyi Lv, Yuchen Zhao, Hailong Liu, Guoling Bi

**Affiliations:** 1 Changchun Institute of Optics, Fine Mechanics and Physics, Chinese Academy of Sciences, Changchun 130033, China; 2 College of Materials Science and Opto-Electronic Technology, University of Chinese Academy of Sciences, Beijing 100049, China

**Keywords:** event cameras, super-resolution, image reconstruction, deep learning

## Abstract

The event camera efficiently detects scene radiance changes and produces an asynchronous event stream with low latency, high dynamic range (HDR), high temporal resolution, and low power consumption. However, the large output data caused by the asynchronous imaging mechanism makes the increase in spatial resolution of the event camera limited. In this paper, we propose a novel event camera super-resolution (SR) network (EFSR-Net) based on a deep learning approach to address the problems of low spatial resolution and poor visualization of event cameras. The network model is capable of reconstructing high-resolution (HR) intensity images using event streams and active sensor pixel (APS) frame information. We design the coupled response blocks (CRB) in the network that are able of fusing the feature information of both data to achieve the recovery of detailed textures in the shadows of real images. We demonstrate that our method is able to reconstruct high-resolution intensity images with more details and less blurring in synthetic and real datasets, respectively. The proposed EFSR-Net can improve the peak signal-to-noise ratio (PSNR) metric by 1–2 dB compared with state-of-the-art methods.

## 1. Introduction

**Figure 1:**
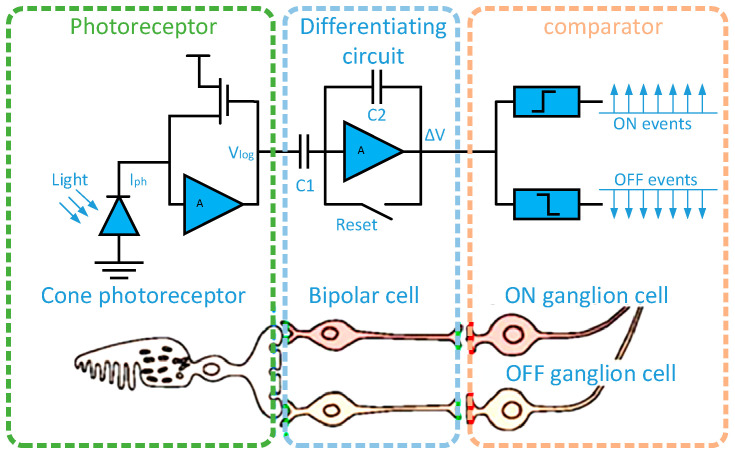
Three-layer model of a human retina and corresponding event camera pixel circuitry. The first layer is similar to retinal cone cells for photoelectric conversion; the second layer, similar to bipolar cells in the retina, is used to obtain changes in light intensity; the third layer is similar to the ganglion cells of the retina for outputting the light intensity change sign.

**Figure 2:**
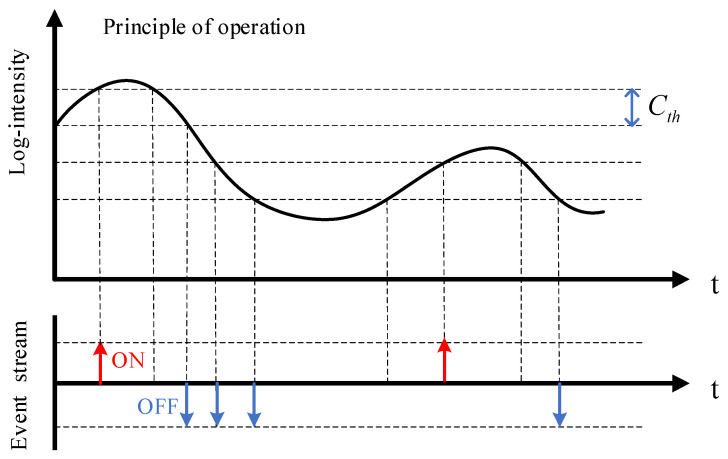
The process of generating events by the event camera. Each pixel acts as an independent detection unit for luminance changes, and events are generated immediately when the log intensity change at the pixel reaches the specified threshold $C_{th}$. Continuous generation of events will form event streams. The event streams contain events of two polarities. When the light intensity changes from strong to weak and reaches the threshold, the camera outputs an OFF event (indicated by the blue arrow); when the light intensity changes from weak to strong and reaches the threshold, the camera outputs an ON event (indicated by the red arrow).

The event camera is a new bio-inspired vision sensor with the pixel structure shown in Figure 1 [1]. The pixel structure is mainly divided into three parts, including a voltage–current logarithmic conversion unit, a change amplification unit and a comparison unit. Event cameras work completely differently than traditional cameras. They do not capture images at a constant rate, but only output information about changes in local pixel brightness. The event generation process is shown in Figure 2 [2]. When the light intensity changes beyond a set threshold, the event camera marks the timestamp with microsecond time resolution and outputs the event. The event information will encode the time, position, and polarity of this change, and the event can be simply represented as a tuple e(x,y,t,p), where *x*, *y* are the coordinates of the output pixel points, *t* is the timestamp of the event, and *p* is the polarity representing the luminance change (polarity information can be defined as a function shown in Equation (1) [3]).

(1) $$p=\{\begin{array}{c} -1\;\;\\ 1 \end{array}\begin{array}{c} if\;OFF\;event\\ if\;on\;event \end{array}$$

A series of events eventually form the event stream, which is shown in Equation (2).

(2) $$E=\overset{N}{\underset{i=1}{\sum }}e\left(x_{i},y_{i},t_{i},p_{i}\right)$$

Due to its unique way of working, the event camera has advantages that traditional cameras do not have, such as low latency, high dynamic range (HDR), high temporal resolution, and low power consumption. Therefore, the event camera is suitable for extreme situations such as high-speed motion and large changes in lighting conditions, making it a research hotspot in robotics and computer vision [4,5,6,7,8].

Considering the need for visualization, the output of the latest dynamic and active pixel vision sensor (DAVIS [6]) combines asynchronous event streams and synchronous image frames. However, on the one hand, the spatial resolution of event cameras is generally not high due to the consideration of data transmission efficiency, i.e., there is a balance between spatial resolution and latency. To meet the low latency data characteristics of event cameras, event cameras are able to capture the luminance changes of each pixel individually [6]. This also leads to its increased noise sensitivity, making the visualization less effective. In summary, reconstructing high-quality intensity images from the output of an event camera and thus meeting the visualization needs of the camera is a significant issue. This will facilitate the application of event cameras to many advanced vision tasks [9,10,11,12], such as recognition, detection, and tracking, that are solved by standard cameras.

Most traditional super-resolution reconstruction methods directly reconstruct intensity images from event stream data [13,14,15,16]. However, the quality of reconstructed images is limited by the sparse nature and low resolution of event stream data, and the intensity information is ignored. Some super-resolution reconstruction methods start to use event data as an aid to super-resolve intensity images. However, due to degradation problems such as blurring and noise for various reasons, they may not produce high-quality images and cannot recover more details in the shadows of real images by using the high dynamic range properties of event data.

In this paper, we propose the EFSR-Net neural network model. It can reconstruct high-quality intensity images with more recovered details and higher dynamic range by using both event data and APS frames as data input. Overall, the main contributions of this paper are as follows:

1. We designed a novel network model suitable for super-resolution reconstruction of intensity images from event cameras, named EFSR-Net. Our algorithm is based on a hybrid paradigm of frames and events. The final super-resolution effect is significantly better than that of simply reconstructing from a low-resolution event stream as input;
2. We designed the coupled response block (CRB) in the network. It can fuse the event data and APS frame data to complement each other, and recover the texture details contained in the real image shadows by using the high dynamic range characteristics of the event data.

The rest of this paper is organized as follows. First, we focus on developing traditional methods for super-resolution reconstruction of event camera images and their respective advantages and disadvantages in Section 2. Secondly, the proposed method is described in Section 3, and the details of our proposed network are systematically presented and analyzed. Next, in Section 4, our experimental part is presented, comparing our method with other state-of-the-art methods and giving qualitative and quantitative analyses to verify the effectiveness of our approach. Finally, conclusions are given in Section 5.

## 2. Related Work

### 2.1. Event Data Processing Method

The event camera converts motion changes into a spatially sparse, temporally dense stream of events. These data contain dynamic information about the edges of the object. However, the event streams are represented in terms of logarithmic intensity changes, which have a completely different data format than the general intensity images [17]. Therefore, the event data are generally processed when reconstruction operations are performed. There have been many approaches to event stream representation and processing, which can be broadly classified into two types based on manual production [15,16,18], and end-to-end [19,20,21]. The primary method of representing events based on manual display uses frequency accumulation images. Frequency accumulation images are divided into fixed-time accumulation images and fixed-number-of-events accumulation images. In the specified time accumulation, the event streams between two consecutive intensity images (APS) of the event camera corresponding to the reference time is divided into n equally scaled parts, forming n frames. These n frames are stacked to eventually form a stack that is fed to the network as input. This approach preserves the temporal dimension information of the event data to a greater extent. However, this stacking method has a problem of missing events when the scene or camera is not moving. When there is not enough event data in the time interval for image reconstruction, it is inevitably difficult to obtain good super-resolution reconstructed images. Also, the number of events in different time intervals may appear excessive. The fixed number of accumulated images is an excellent way to avoid the above problem. It forms frames by merging events according to the number of incoming events. The first $N_{e}$ events are merged into frame 1, the next $N_{e}$ events are merged into frame 2, and so on, creating n frames that form a stack. Since we count the number of events over time, we can adaptively adjust the number of events in each frame as well as in a stack.

End-to-end event representation methods mainly include grid-based representation, which samples event streams into a spatial–temporal voxel grid, and point-set-based representation, which is treated as a point cloud. They can both use end-to-end neural networks, and supervised learning driven by big data can better mine the spatial–temporal properties of event stream signals. However, these types of methods cannot manipulate the data and cannot select well near the timestamp we are interested in.

### 2.2. Event-Based Intensity Reconstruction

Due to its wide range of applications, the reconstruction of intensity images from events has become a hot research topic in event cameras. An earlier attempt to study intensity reconstruction from pure events was to consider relatively short-period event stream data and directly accumulate positive and negative events of two colors as the output of the gradient interpretation. Hanme Kim et al. proposed to replace the gradient representation with a synthetic intensity image using simultaneous estimation of camera motion and stitching it into a panoramic gradient image. In this method, the scene is static, with only rotational motion of the camera. The gradient image is finally converted to an intensity image by Poisson integration [20]. The reconstruction of HR images based on spherical 3D scenes was further investigated in depth in [12]. In [21], Cook et al. proposed a cyclic structured bionic interconnection network that achieves simultaneous reconstruction of intensity frames, optical flow, and angular velocity of small rotational motions. Bardow et al. [22] proposed the joint estimation of intensity variations and optical flow in a unified variational energy minimum framework in a challenging dynamic motion environment. The optimization allows simultaneous reconstruction of video frames and optical streams, but this method propagates errors as shadow-like artifacts in the generated intensity images. A variational framework based on a denoising scheme that iteratively filters incoming events was introduced in [23]. The method uses a popular regularization on the relative timestamps of the events to recover more details in texture-free regions to achieve reconstructed images. In [14], a high-pass filter was proposed to reconstruct the video in a very efficient way. The framework was originally designed to complement intensity frames with event information, but can also reconstruct images from events without the help of APS frames. In contrast to [14], Munda et al. [23] considered image reconstruction as defined in the energy minimization problem induced by the event timestamp. In recent years, deep learning-based methods have greatly progressed in intensity image and video reconstruction. Wang et al. [15] took to reconstructing intensity images using generative adversarial networks on the U-Net [24] architecture. Rebecq et al. [13] used recurrent neural networks and stacked ConvLSTM gates to reconstruct videos from events. The above methods all perform intensity reconstruction from event stream data, while APS frames contain relatively rich textures, so events and APS frames can be used as complementary sources for event-based intensity reconstruction. In [14], events are approximated as temporal differences in intensity frames. On this basis, a complementary filter is proposed as a fusion engine that can generate intensity frames almost continuously. Pan et al. [25] proposed an event-based deblurring method by correlating blurred APS frames and events with an event-based double integration (EDI) model. Next, a multi-frame EDI model for high-speed video reconstruction is proposed by further considering the relationship between frames [26]. Zhang et al. [27] formulated event-based image reconstruction as a linear inverse problem with deep regularization using optical flow. They have emphasized the framework of simultaneously estimating both physically entangled quantities in the events: brightness and motion (optical flow).

### 2.3. Event-Based Super-Resolution

Since the spatial resolution of event cameras is limited, some work in recent years has focused on the super-resolution of event streams in the spatial and temporal domains. Mohammad et al. [16] were the first to design recurrent neural networks to solve the super-resolution event camera problem. They created an SRNet network to perform super-resolution reconstruction from pure event data. Li et al. [28] used Poisson point processes to model event sequences and sampled the events according to a nonhomogeneous Poisson process. Duan et al. [29] proposed EventZoom, which is a deep neural network framework with a three-dimensional U-Net [24] backbone architecture that addresses joint denoising and super-resolution of neuromorphic events. Wang et al. [18] proposed an end-to-end network called EventSR, which implements image reconstruction, enhancement, and upsampling from the event streams. The network is trained in three stages and uses unsupervised adversarial learning. They also created an open dataset. Wang et al. [30] designed a sparse learning network for event enhancement to simultaneously solve the deblurring, denoising and super-resolution problems. In [31], a hybrid camera was constructed to guide the event filtering and take advantage of the HR RGB signal to guide the upsampling of events. In [17], Han et al. converted event data into potential intensity frames and reconstructed SR intensity images and high frame rate videos with higher dynamic range and less blurring artifacts by fusing potential frames with APS frames. Weng et al. [32] proposed a recurrent neural network for event SR without the assistance of frames. They also demonstrated the feasibility of achieving event SR solely with pure events. Song et al. [33] proposed E-CIR, which converts a blurry image into a sharp video represented as a parametric function from time to intensity. It achieves good results in deblurring but still lacks in improving image resolution.

## 3. Proposed Method

### 3.1. Overview

**Figure 3:**
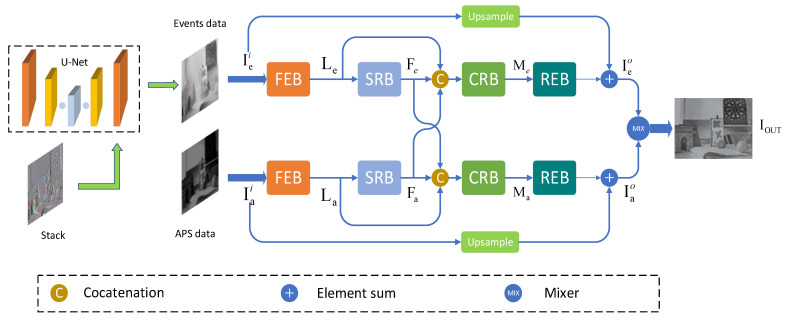
EFSR-Net network structure. The event data is first preprocessed to form a stack, followed by a series of encoding and decoding through the network. The processed event information and APS frame information are used as inputs into the upper and lower coupling sub-networks. Each sub-network consists of a feature extraction block (FEB), a coupled response block (CRB), and a reconstruction block (REB). The final super-resolution image reconstruction is achieved by the mixer (MIX) convolutional network.

In this paper, we design a convolutional neural network EFSR-Net consisting of several modules. Figure 3 shows the overall structure of our proposed EFSR-Net. Our network takes mixed types of data as input and fuses intensity frame information with event stream information to achieve super-resolution reconstruction of high-quality intensity images from event cameras. Our approach is divided into two main steps. The event stream is first preprocessed with data, and we choose event stacking for processing, as in [16]. This method is able to select event data near the timestamp we are interested in. In the second step, the APS frames and the stack containing the event information are passed through the neural network as input for super-resolution reconstruction. Our super-resolution network consists of upper and lower coupled sub-networks for feature extraction on event frames and APS frames, respectively. In particular, we designed a coupled reaction block (CRB) to achieve the fusion and complementarity of the two data features. In Section 3.2, we describe the preprocessing method for event data. In Section 3.3, the network structure is introduced, explaining the details of the whole network architecture. In Section 3.4, the loss function of our network is described.

### 3.2. Event Data Preprocessing

The event streams data has HDR properties and high temporal resolution. We aim to perform intensity image super-resolution reconstruction using APS frames combined with event data around the corresponding timestamps. Event streams represent sparse logarithmic intensity changes and have a completely different type of data format than ordinary intensity images. Therefore, it is not easy to fuse event data and intensity image information directly. In [34], it is stated that under ideal conditions (noise-free scenes, perfect sensor response, etc.), the integration of events produces “absolute” luminance, i.e., it is possible to integrate intensity images from event information, since events simply encode the visual content of the scene in a non-redundant way for each pixel. In addition, due to the high temporal resolution of the events, the luminance images can meet a very high frame rate for reconstruction [14].

Since we need to obtain the event stream data around the corresponding APS frame, as mentioned earlier, end-to-end event processing methods based on grids, etc., are not suitable. Therefore, this paper uses a stacking approach based on the number of events, where a fixed number of events are stacked to form a stack. The specific stacking method is to start counting the number of events according to the timestamp information until the predefined number of events $N_{e}$ is reached, and the accumulated events form a channel in the stack. The above work is repeated *C* times to form *C* channels. We make the size of the stack consistent with the size of the APS frame and finally obtain some column stacks $S_{i}\in {\mathbb{R}}^{H\times W\times C},\;i=1,\;2,\;3,\ldots$. Therefore, each stack contains *M* = *C* × $N_{e}$ events. In this paper, we set *C* = 3, $N_{e}\;$ = 5000, *H* = 320 and *W* = 240.

### 3.3. Network Architecture

Inspired by [15], we used the basic architecture of U-Net [24] to finally include our event data in the intensity image through an encoder and decoder to facilitate the processing of event data by our subsequent network. We performed a series of encoding and decoding as well as hopping connections on the stack $S\in {\mathbb{R}}^{H\times W\times C}$ formed by stacking events, and finally output the intermediate intensity image $I_{e}^{i}\in {\mathbb{R}}^{H\times W\times 1}$. Next, we fed the intermediate intensity image data containing the event data and the APS frame data to our super-resolution network. Our super-resolution network consists of two coupled sub-networks, upper and lower, taking the processed event data and the APS frame image data as input, respectively. Each sub-network contains an initial feature extraction block (FEB), a super-resolution block (SRB), a coupled response block (CRB), and a reconstruction block (REB). The APS frame data is denoted as $I_{a}^{i}$. The corresponding features extracted by FEB can be expressed as:

(3) $$L_{e}=f_{FEB}\left(I_{e}^{i}\right)$$

(4) $$L_{a}=f_{FEB}\left(I_{a}^{i}\right)$$

where $f_{FEB}$ indicates the operation of the feature extraction block.

The FEB uses a convolutional layer with PReLU activation, consisting of 256 filters sized 3 × 3 precisely, to extract the features of the image. The extracted features $L_{e}$, $L_{a}$ are used as the basic inputs for the subsequent SRB and CRB. Using the basic features $L_{e}$, $L_{a}$ as input, the role of the SRB is to learn more advanced features and improve the image resolution. The feedback architecture of the SRB follows [35]. The advanced features learned by the SRB can be expressed as:

(5) $$F_{e}=f_{SRB}\left(L_{e}\right)$$

(6) $$F_{a}=f_{SRB}\left(L_{a}\right)$$

where $f_{SRB}$ denotes the operation of the SRB. $F_{e}$ and $F_{a}$ denote the intermediate intensity image containing event information and the advanced features extracted from the APS frame image, respectively, which are important inputs for the subsequent CRB. 

The coupled response block (CRB) enables simultaneous super-resolution and fusion of information from two data sources through complex network connections. We take the outputs $F_{e}$ and $F_{a}$ of the SRB module and the features $L_{e}$ and $L_{a}$ extracted at the FEB as inputs, first connected by a set of 1 × 1 filters, and then perform a series of upsampling and downsampling operations to extract the feature map. The output $F_{e}$, $F_{a}$ of the SRB module is used to correct the basic characteristics of $L_{e}$, $L_{a}$ and improve the performance of SR. Furthermore, $F_{e}$, $F_{a}$ bring complementary information to each sub-network to achieve feature fusion. The output can be expressed as:

(7) $$M_{e}=f_{CRB}\left(F_{e},F_{a},L_{e}\right)$$

(8) $$M_{a}=f_{CRB}\left(F_{a},F_{e},L_{a}\right)$$

where $f_{CRB}$ denotes the operation of the CRB. $M_{e}$, $M_{a}$ are the outputs of the CRB blocks in the upper and lower sub-networks. 

After the data stream passes through the upper and lower two CRBs, the information is reconstructed in the next step. The reconstruction can be expressed as follows:

(9) $$I_{e}^{o}=f_{UP}\left(I_{e}^{i}\right)+f_{REB}\left(M_{e}\right)$$

(10) $$I_{a}^{o}=f_{UP}\left(I_{a}^{i}\right)+f_{REB}\left(M_{a}\right)$$

here $I_{e}^{o}$ and $I_{a}^{o}$ are the intermediate output intensity information of the upper and lower subnetworks, respectively. $f_{UP}$ denotes the upsampling operation and $f_{REB}$ denotes the reconstruction block operation. 

Finally, the reconstructed features are fused by our mixer (Mix) using convolutional layers to finally generate super-resolution intensity images. The output can be expressed as follows:

(11) $$I_{out}=w_{e}I_{e}+w_{a}I_{a}$$

where $I_{out}$ is the reconstructed image we finally obtain, and the initial values of $w_{e}$ and $w_{a}$ are chosen as 0.5.

### 3.4. Loss Function

There are many choices of loss functions used for optimization, such as L1 loss function, L2 loss function, perceptual loss function, perceptual similarity loss function, and so on. Because the L2 loss function causes the output image to become smooth, we choose the L1 loss function in order to get a clearer image. However, the L1 loss can only characterize the low-level feature differences of the image, which can easily cause the image not to match the visual perception of the human eye. So we add perceptual similarity loss [36] to the L1 loss function as in [16]. We extract the feature stack from layer l and perform unit normalization in the channel dimension, and the perceptual similarity loss is calculated as follows:

(12) $$L_{ps}=\underset{l}{\sum }\frac{1}{H_{l}W_{l}}\underset{h,w}{\sum }∥w_{l}⨀{\left({\hat{y}}_{hw}^{l}-{\hat{y}}_{0hw}^{l}\right)∥}_{2}^{2}$$

where ${\hat{y}}^{l},{\hat{y}}_{0}^{l}\in {\mathbb{R}}^{H_{l}\times W_{l}\times C_{l}}$ denotes the *l*-layer channel dimension. The vector $w_{l}\in {\mathbb{R}}^{C_{l}}$. The total loss function is:

(13) $$L_{loss}=L_{1}+\lambda L_{ps}$$

where λ is the balance parameter. The network is trained by minimizing the loss function to bring the output closer to the desired image.

## 4. Experiment

### 4.1. Dataset Preparation 

We need APS frame and event stream data and the corresponding ground truth image sequences for network training. However, the dataset collected by the real event sensor can only provide low-resolution images of poor quality, which cannot be used as the ground truth images needed for training. Therefore, we trained the network on a synthetic dataset we borrowed from [30] and processed it. It selects clear images with a resolution of 1280 × 720 from the Gopro dataset [37] as the ground truth (GT) images and generates events from a series of input images using the event camera simulator (ESIM) [38]. We choose 120 video sequences, each containing 95 images, for a total of 11,400 images. We generate the corresponding low-resolution images in our experiments by sampling HR images with bicubic interpolation. Their resolutions are 640 × 360 and 320 × 180, corresponding to our 4× and 2× training, respectively. In the experiments, 80% of the dataset is used for training and 20% for testing.

### 4.2. Implementation Details 

During the training process, the initial learning rate is set to 0.01, the total number of training units is 80, and the balance parameter λ is 0.3. After 50 epochs, the learning rate decays to 0.001, and the batch size is set to 8. The optimizer uses the Adam [39] algorithm with parameters $\beta_{1}$ = 0.9 and $\beta_{2}$ = 0.999.

We implemented our proposed model in Pytorch version 1.12.0 and Python version 3.9, which were trained using a single NVIDIA GeForce RTX 3090 GPU. the CUDA and CuDNN versions are 11.6 and 8.4.0, respectively.

### 4.3. Compare with Advanced Algorithms

In this work, we compare the super-resolution algorithm with state-of-the-art event cameras on synthetic and real datasets, respectively, to demonstrate the superiority of our approach and its generalizability to real scenarios. To my knowledge, there are only a few super-resolution algorithms for event cameras, so, like other related articles [16,17], we also compare the reconstruction algorithm for event cameras combined with the image-based super-resolution algorithm. The comparison is as follows:

1. EV [15] + SISR [40], the method first reconstructs the intensity image by E2VID [15] using the event streams data as input and then super-resolves it with the trained single image super-resolution (SISR) network [40];
2. E2SRI [16], the method uses pure event data as input to super-resolve the reconstructed intensity image;
3. eSL-Net [30], which uses APS frames and event data to reconstruct HR intensity images super-resolved.

We demonstrate the superiority of our proposed method from both qualitative and quantitative aspects, respectively. For the quantitative analysis of the experiments, we choose peak signal-to-noise ratio (PSNR) and structural similarity (SSIM) as our evaluation metrics. The following is a detailed description of the evaluation metrics:

The peak signal-to-noise ratio (PSNR) can be expressed as:

(14) $$PSNR\left(Y,\hat{Y}\right)=10\log_{10}\left(\frac{\max{\left(Y\right)}^{2}}{MSE\left(Y,\hat{Y}\right)}\right)$$

where $\hat{Y}$ represents the generated image and *Y* represents the original image, MSE is the mean squared error; the larger the PSNR, the better the quality of the image.

The structural similarity (SSIM) is a measure of how similar two images are, and can be expressed as:

(15) $$SSIM\left(Y,\hat{Y}\right)=\frac{\left(2\mu_{Y}\mu_{\hat{Y}}+c_{1}\right)\left(2\sigma_{Y\hat{Y}}+c_{2}\right)}{\left(\mu_{Y}^{2}+\mu_{\hat{Y}}^{2}+c_{1}\right)\left(\sigma_{Y}^{2}+\sigma_{\hat{Y}}^{2}+c_{2}\right)}$$

where $\mu_{Y}$ is the mean of *Y*, $\mu_{\hat{Y}}$ is the mean of $\hat{Y}$, $\sigma_{Y}^{2}$ is the variance of *Y*, $\sigma_{\hat{Y}}^{2}$ is the variance of $\hat{Y}$, and $\sigma_{Y\hat{Y}}$ is the covariance of *Y* and $\hat{Y}$. $c_{1}={\left(k_{1}L\right)}^{2}$, $c_{1}={\left(k_{2}L\right)}^{2}$ are constants used to maintain stability. *L* is the dynamic range of pixel values. $k_{1}=0.01$, $k_{2}=0.03$.

#### 4.3.1. Evaluation on Synthetic Datasets

In this section, we use both qualitative and quantitative methods to illustrate the effectiveness of our proposed method in terms of both visual perspective and objective evaluation metrics. Our test set consists of 2280 intensity images and a stream of events between two consecutive frames from 24 high-frame rate video sequences. The quantitative results (PSNR, SSIM) are shown in Table 1, and our method is highly competitive with other popular methods. It should be noted that in performing the comparison, the E2SRI [16] method does not have a 4× SR pre-trained model, so the PSNR and SSIM values are not available in the corresponding positions in Table 1. The eSL-Net [30] method does not have a pre-trained model of 2× SR, and we use bicubic interpolation to process its 4× SR results to obtain the quantitative results corresponding to 2× SR. Compared to the above methods, it can be seen from the quantitative evaluation that our approach is able to reconstruct higher quality 2× and 4× high-resolution intensity images in synthetic dataset experiments. Furthermore, the fusion of intensity images and event data can achieve higher quality image super-resolution with more structural details than reconstructing images from event data alone.

**Figure 4:**
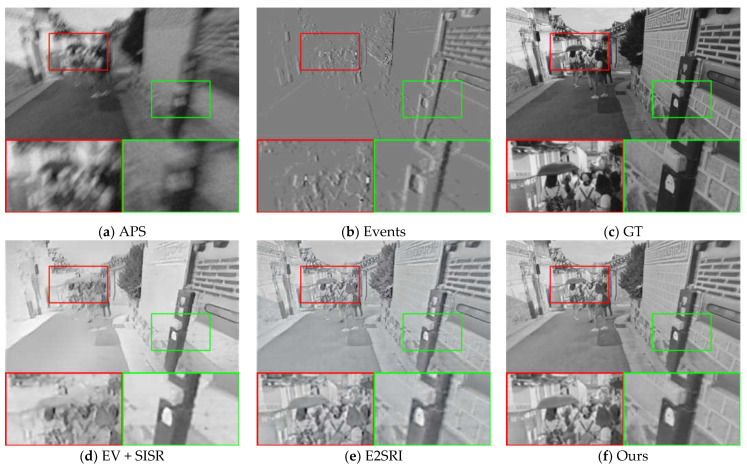
Comparison of the visual quality of our proposed method with other state-of-the-art methods for 2× SR on synthetic datasets. The APS frame and event stack are upsampled with bicubic interpolation to the corresponding scale for reference.

**Figure 5:**
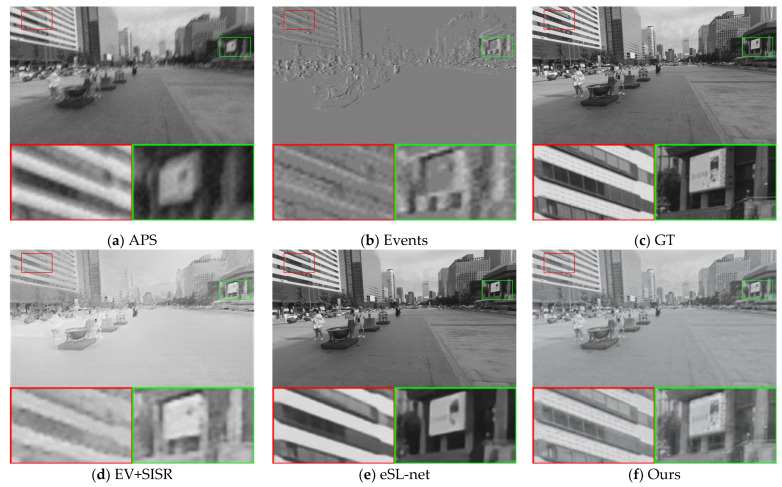
Comparison of the visual quality of our proposed method with other state-of-the-art methods for 4× SR on synthetic datasets. The APS frame and event stack are upsampled with bicubic interpolation to the corresponding scale for reference.

Figure 4 and Figure 5 visualize our comparison results with other methods at 2× SR and 4× SR, respectively, from a visual perspective. A comparison of the reconstruction quality of the different methods in the two scenes from the synthetic dataset shows that the event data contains more edge detail information in the scene. A comparison of the APS frames with the event stacked images in Figure 4 shows that the event data capture more information about the scene and is unaffected by blurring. Comparing the EV [15] + SISR [40] method in Figure 4 with our method, it can be seen that our method is able to reconstruct high-quality images with more continuous grayscale. This indicates that the APS frames provide more continuous grayscale information for our image reconstruction, which complements the drawback of too little event data in scenes with little change in light intensity and thus distortion of the reconstructed image.

#### 4.3.2. Evaluation on Real Dataset

**Figure 6:**
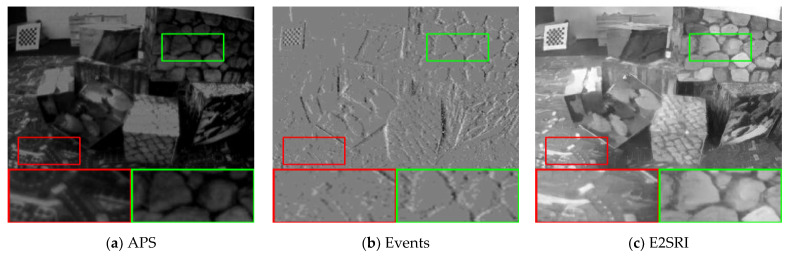

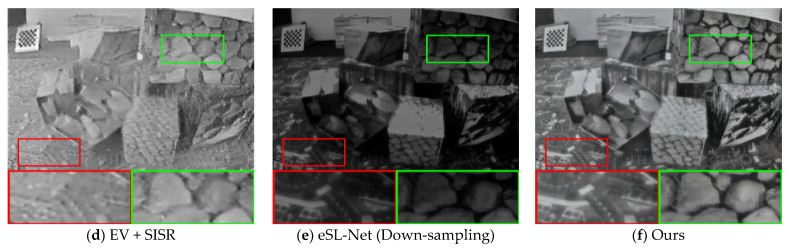
Comparison of the visual quality of our proposed method with other state-of-the-art methods for 2× SR on real datasets. The APS frame is upsampled with bicubic interpolation to the corresponding scale for reference. The 4× SR results of eSL-Net is downsampled with bicubic interpolation to the corresponding scale for reference.

**Figure 7:**
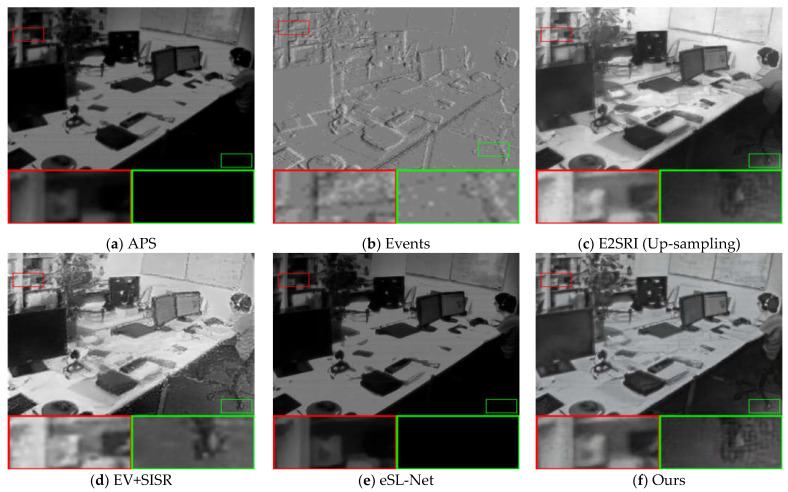
Comparison of the visual quality of our proposed method with other state-of-the-art methods for 4× SR on real datasets. The APS frame and the 2× SR results of E2SRI are upsampled with bicubic interpolation to the corresponding scale for reference.

**Figure 8:**
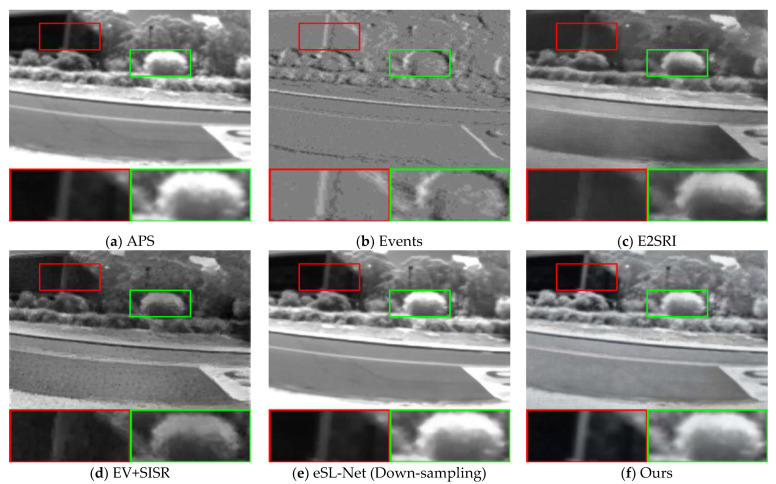
Comparison of the visual quality of our proposed method with other state-of-the-art methods for 2× SR on real datasets. The APS frame is upsampled with bicubic interpolation to the corresponding scale for reference. The 4× SR results of eSL-Net is downsampled with bicubic interpolation to the corresponding scale for reference.

To further illustrate the effectiveness of our proposed algorithm, we compared our method with other methods on seven sequences from the ESIM real dataset [41] as well as sequences from [14]. These sequences were recorded using a DAVIS240C sensor [6] moving in various environments. It contains events as well as grayscale frames at a rate of 20 Hz. Since there are no corresponding high-quality images in the real dataset that can be used as ground truth, we performed qualitative analysis on three sequences of boxes_6dof, office_zigzag, and motorbike from different datasets, and the results are shown in Figure 6, Figure 7 and Figure 8.

As with the comparison method for the synthetic dataset, we compare the 2× SR and 4× SR results of the different methods on sequences of the real dataset separately, and the SR results demonstrate that our method is able to reconstruct more details and sharper edges. Compared to the results on the synthetic dataset, in the real dataset, our method results in images with a higher dynamic range and is able to recover the texture details contained in the shadows of the real images. This is made possible by the high dynamic range nature of the event camera, which allows the event data to contain more information about the scene. From the visual viewpoint, it can be seen that our method has the best visual effect in the real dataset as well. This shows that our method is equally applicable in real scenarios.

### 4.4. Ablation Experiment

**Figure 9:**
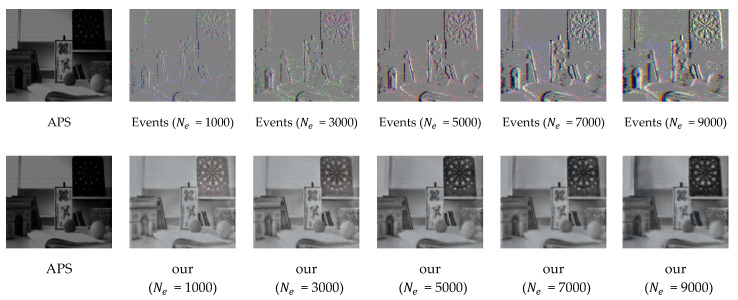
The effects of different values of $N_{e}$ on event stacks and reconstructed images are qualitatively compared. The APS frame is upsampled with bicubic interpolation to the corresponding scale for reference.

To demonstrate the effectiveness of the perceptual loss function added to our loss function, we conducted ablation experiments by removing the perceptual loss function from the total loss function. From Table 2, we can see that we can improve our evaluation metrics by introducing the perceptual loss function in the loss function, which proves its effectiveness. We choose the slider_depth sequence of the ESIM real dataset [36] to conduct ablation experiments on the variable $N_{e}$ that controls the number of events. This further proves the effectiveness of our method. Our aim is to reconstruct the intensity image with higher resolution and recover more details, so we show the effect of changing $N_{e}$ on the reconstruction quality from a visual perspective, and the results are shown in Figure 9. It can be seen from the figure that a small number of events in the stack will make the event flow information too small to provide more details of the scene, resulting in low reconstruction quality; too many events in the stack will make subsequent events overwrite previous events, resulting in blurring and smearing, resulting in low reconstruction quality.

## 5. Conclusions

In this paper, we propose a novel network called EFSR-Net for reconstructing high-resolution intensity images from event cameras, which addresses the problem of the low spatial resolution of event cameras. In particular, we propose that the coupled response block (CRB) is able to fuse the event streams with the feature information of APS frames to complement each other. We exploit the high dynamic range properties of the event stream data to enable the reconstructed images to recover the texture details contained in the shadows of the real images. Our experiments on synthetic and real datasets demonstrate the superiority of EFSR-Net, and that EFSR-Net outperforms existing methods in terms of qualitative and quantitative results.

## Figures and Tables

**Table 1:** Quantitative evaluation of 2× and 4× SR. ↑ means the higher (lower), the better results throughout this paper.

Scale	Method	PSNR ↑	SSIM ↑
2×	EV [15] + SISR [40]	12.52	0.466
	E2SRI [16]	16.41	0.587
	eSL-Net [30]	15.76	0.534
	Ours	22.02	0.746
4×	EV [15] + SISR [40]	11.93	0.572
	E2SRI [16]	-	-
	eSL-Net [30]	21.84	0.683
	Ours	23.25	0.714

**Table 2:** Ablation study of the loss function.

Loss	PSNR	SSIM
L1	21.98	0.698
$L_{loss}$	22.02	0.746

## Data Availability

Not applicable.
